# Sorption of pentachlorophenol (PCP) in the marine bottom sediments—batch sorption experiment at varying pressure

**DOI:** 10.1007/s11356-017-1076-x

**Published:** 2018-02-02

**Authors:** Krzysztof Konrad Lewandowski, Witold Cieślikiewicz, Marta Ewelina Kobusińska, Elżbieta Niemirycz

**Affiliations:** Institute of Oceanography, University of Gdańsk, Al. Piłsudskiego 46, 81-378 Gdynia, Poland

**Keywords:** Hydrostatic pressure, Microcosm, POPs, Sand and fine sand, Harbor sediments, The southern Baltic Sea

## Abstract

Study was undertaken to determine the effect of hydrostatic pressure on the sorption of pentachlorophenol (PCP). The experiment was conducted at atmospheric pressure (1000 hPa) and at increased pressure (6000 hPa) simulating conditions at the water depth of 50 m. The sorption of PCP was examined in an artificial environment (microcosm) consisting of the marine water and the bottom sediments from a Polish harbor and the southern Baltic Sea. The first part of the experiment comprised the determination of PCP sorption parameters in the microcosms and parameters of the sediments (organic matter content, conductivity) and of the overlying water (pH, ion concentration) at 1000 hPa. The second part of the experiment was conducted at 6000 hPa inside the hyperbaric chamber. The hyperbaric exposure affected parameters of the harbor sediments and the overlying water but had little influence on the concentration of PCP in the microcosms containing the southern Baltic Sea sediments. Considering the specific characteristics of the harbor sediments, it can be assumed that the impact of hydrostatic pressure on the sorption process of PCP at 50-m depth appears to be negligible.

## Introduction

Sorption and desorption processes, occurring in the seabed, are important in the marine environmental studies because they affect the composition of marine water and also transport and transformation of chemicals (Hedman et al. 2008; Soubaneh et al. 2015). The effect of hydrostatic pressure on the life processes of bacteria population was examined by numerous researchers (Somero 1992; Schedler et al. 2014; Scoma et al. 2016) but studies on the effect of hydrostatic pressure on sorption of contaminants in sediments have not been undertaken. Typically, sorption experiments are carried out at atmospheric pressure. Therefore, the possible influence of high pressure caused by a water column, i.e., hydrostatic pressure, is not taken into consideration. This force, depending on the water depth, can attain high values and possibly influence the sorption/desorption processes. One of the objectives of this study was to determine whether, and eventually by how much, a pressure increase of 5000 hPa can affect these processes.

Persistent organic pollutants (POPs), including pentachlorophenol (PCP) and its salts and esters, belong to the hydrophobic compounds (The Stockholm Convention 2015). This substance was added to the Stockholm Convention on the basis of adverse effects on humans and animals health, molar mass more than 236 g mol^−1^ (increased toxicity), the water/octanol partition parameter within 5.0 (a significant bioaccumulation), and large-scale production (U. S. Department of Health and Human Services 2014). Strong sorption process can effectively affect the removal of a substance from the water and immobilize it on/in grains forming the bottom. However, as a result of bioturbation, influence of current fields and changes in pH or temperature desorption followed by secondary pollution can occur (Chorover and Brusseau 2008; Hedman et al. 2009; Maciak et al. 2016).

Sorption is the phenomenon of substance (sorptive) accumulation at the surface (adsorption) of a solid (sorbent) and the phenomenon of uptake of the sorptive by entire sorbent (absorption). Sorption experiments are carried out using two-phase systems: solid-liquid, gas-liquid, or solid-gas. These experimental systems, designed to reflect in a smaller-scale processes occurring in the environment, are called microcosms (Hedman et al. 2008; Olsen et al. 2017). When microcosm consists of solid (I)–liquid (II) system, the matrix can be composed of (I) natural bottom sediments or only its selected fraction (sand, silt), soil, mineral (montmorillonite), and various types of biomasses; and (II) marine or freshwater, deionized water, or artificially created water on the basis of typical electrolytes (CaCl_2_, MgCl_2_) with the addition of a buffer solution (Chen et al. 2004; Subramanian et al. 2010; León-Santiesteban et al. 2014; Liping et al. 2015).

To describe the environmental activity and the parameters of given compounds, computer modeling can be applied, for example, quantitative structure-activity relationship (QSAR) method (Puzyn et al. 2008). The source of contaminants can also be detected by statistical methods (Witt 2015).

This study aimed to determine the differences in the kinetics of geochemical processes at room temperature under atmospheric pressure (about 1000 hPa) and pressure increased to 6000 hPa. The conditions in microcosms at the raised pressure corresponded to the conditions at a water depth of 50 m. The research hypothesis states that force of increased hydrostatic pressure at a depth of 50 m should accelerate sorption process of pentachlorophenol on sediments, compared to its speed at atmospheric pressure.

## Materials and methods

### Bottom sediment sample area—study area

**Fig. 1:**
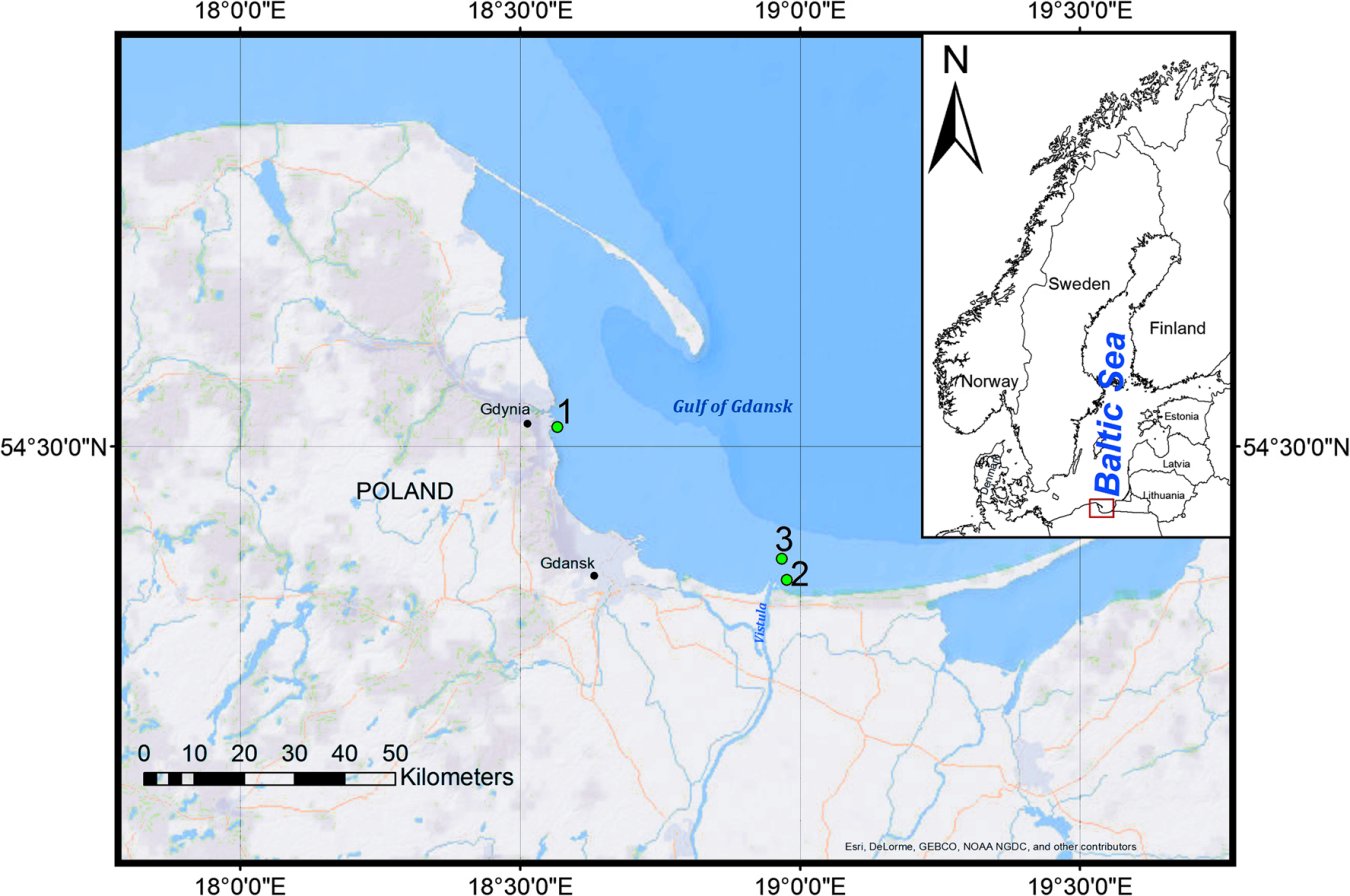
Sampling sites of bottom sediments from the Port of Gdynia (1) and bottom sediments (2) and water (3) from the Gulf of Gdańsk

The solid phase, i.e., top 5 cm of bottom sediments/sorbents, were collected in 2012 with a Van Veen grab from a depth of 10 m from the Port of Gdynia and in 2014 from a depth of 40 m from the area under influence of the Vistula River waters in the Gulf of Gdańsk (Fig. 1). The main types of harbor sediments are fluvioglacial sands, boulder clays, and silty clays (Dworniczak et al. 2017). The sediment types in the vicinity of the river (sample point 2) are fine sands and sandy silts. The sediments were air-dried, homogenized, and sieved through a 2-mm-diameter mesh sieve, and visible parts of plants, coal, bricks, and the like were removed. Before the experiments, performed in the years 2015–2016, sediments were kept in a freezer at − 21 °C.

### Water sample area

The liquid phase, i.e., marine water, was sampled in 2015, from the Gulf of Gdańsk (Fig. 1) from a depth of 0.5 m using Teflon bottles and was aged for 6 months in room temperature for natural organic matter degradation. Typical salinity in the area of the Gulf of Gdansk is from 7.0 PSU around the Port of Gdynia to 3.0 PSU in the vicinity of the Vistula River mouth.

Before conducting the experiments, selected parameters of the bottom sediments (Table 1) and the water (Table 2) were determined after filtration through a GF/F filter.

**Table 1:** Physicochemical parameters of bottom sediments used in the study

Station	Sand fraction [%]	Fine fraction *Φ* < 0.063 mm [%]	^a^PCP [ng g^−1^ d.w.] ± SD	TOC [%] ± SD	LOI [%] ± SD	Conductivity [mS cm^−1^] ± SD	Sediment type
Port of Gdynia	78.80	21.20	25.0 ± 3.60	2.37 ± 0.10	5.20 ± 0.59	5.30 ± 0.09	Sandy silt
Gulf of Gdańsk	99.10	0.90	< LOD	0.34 ± 0.02	0.70 ± 0.10	0.40 ± 0.03	Medium sand

*TOC* total organic carbon, *LOI* loss on ignition, *SD* standard deviation, *LOD* limit of detection

^a^Maciak et al. (2016)

**Table 2:** Physicochemical parameters of water used in the study

Station	PCP [ng cm^−3^] ± SD	Cl^−^ [mg dm^−3^] ± SD	Σ of Ca^2+^ and Mg^2+^ [mg dm^−3^] ± SD	Conductivity [mS cm^−1^] ± SD	pH ± SD
Gulf of Gdańsk	NDA	3220 ± 40	412 ± 19	11.30 ± 0.05	7.90 ± 0.01

*SD* standard deviation, *NDA* no data available

### Pentachlorophenol—sorptive

The solution of pentachlorophenol (PCP, CAS number 87-86-5) added to the marine water in the microcosm was prepared from standard solution (99.9%, Sigma-Aldrich, Poland) diluted with methanol to form a 5-μg-cm^−3^ stock solution. Development of microorganisms in the liquid phase was limited by adding sodium azide (NaN_3_) at a final concentration of 200 mg dm^−3^ (Chen et al. 2004).

### Experimental setup at atmospheric pressure

The first part of the experiment was conducted at atmospheric pressure, at ambient temperature, and in the dark. The microcosm consisted of a 125-ml glass jar in which 20 g of the bottom sediments were placed and filled with marine water (Table 2), PCP, and NaN_3_ solutions in final volume of 40 ml. The control sample, done in triplicate, consisted of marine water, PCP, and NaN_3_ solution only.

Three sorbent types were prepared from the harbor sediments: *Sandy* (*S*)—grain size between 2.00 and 0.125 mm separated by mechanical shaking, *Fine* (*F*)—*S* sorbent with approximately 25% of grain size < 0.063 mm separated by mechanical shaking, and *Ignited* (*I*)—*S* sorbent ignited at 450 °C in a muffle furnace (Table 3). The microcosms with *S*, *F*, and *I* sorbents were done in four replicates. All microcosms were stabilized at atmospheric pressure for 1 day. Afterwards, 1 ml of PCP stock solution was added to liquid phase and the experiment was conducted for 8 and 24 h.

**Table 3:** Physicochemical parameters of sediments collected from the Port of Gdynia

Sorbent	PCP [ng g^−1^ d.w.] ± SD	TOC [%] ± SD	LOI [%] ± SD	Conductivity [mS cm^−1^] ± SD	pH ± SD
*Sandy* (*S*)	< LOQ	1.11 ± 0.03	3.10 ± 0.15	2.52 ± 0.04	7.10 ± 0.01
*Fine* (*F*)	12.50 ± 1.10	1.92 ± 0.14	5.90 ± 0.35	6.18 ± 0.08	7.22 ± 0.01
*Ignited* (*I*)	< LOQ	0.42 ± 0.01	0.90 ± 0.07	6.04 ± 0.06	7.01 ± 0.02

*TOC* total organic carbon, *LOI* loss on ignition, *SD* standard deviation, *LOQ* limit of quantification

Bottom sediments from the Gulf of Gdańsk (Table 1) were used to prepare two sorbent types: *Natural I* (*N I)—*unmodified sediments from point 2 (Fig. 1) and *Natural II* (*N II*)*—*modified *N I* sorbent by adding approximately 25% of grain size < 0.063 mm separated by mechanical shaking, giving loss on ignition (LOI) of 3.4%. After initial exposure of *N I* (6 replicates) and *N II* (3 replicates) microcosms to 6000 hPa over 24 h, 1.6 ml of PCP stock solution was added to liquid phase and the sorption experiment at 1000 hPa was conducted for 8 h. In the following discussion, sorbent name is also name of microcosm.

### Experimental setup at increased pressure

**Fig. 2:**
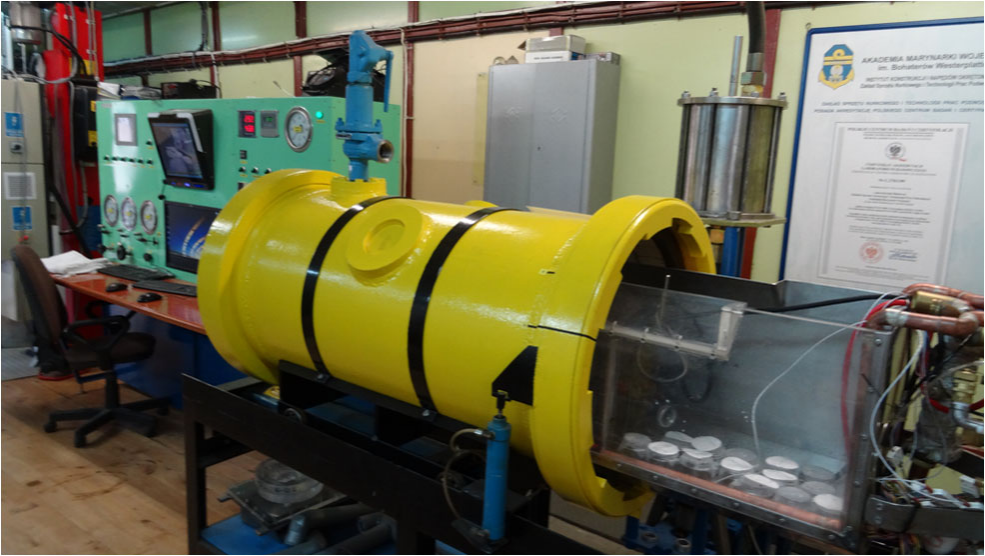
The hyperbaric chamber (the Department of Underwater Works Technology of the Polish Naval Academy in Gdynia)

The experiment at 6000 hPa was conducted in the hyperbaric chamber of the Polish Naval Academy (PNA) in Gdynia (Fig. 2) keeping the same sorbent setup as for the experiment performed at atmospheric pressure. The required parameters were set via the control panel. The pressure was obtained by pumping atmospheric air into the chamber.

After each experiment, the overlying water was filtered through a combusted GF/F filter to glass vials fitted with Teflon caps. All the laboratory work was done at the Institute of Oceanography of the University of Gdańsk. The sorbents were dried at ambient temperature for 24 h and, after that, at 105 °C for 6 h. The water samples were kept at 7 °C for 12 h to 1 week and sorbents at − 21 °C for 1 week to 1 month.

In all the water samples and all the sediment samples, the fallowing measurements were taken: conductivity (*n* = 3)—with a Multi 3320 (WTW) and pH (*n* = 3)—with a pH meter (Elmetron). In the case of sorbents, measurements were done by pouring 30 ml of Milli-Q water onto 10 g of air-dried sediments and shaking for 1 h to reach stable reading. The concentration of Cl^−^ anion (Mohr’s method, K_2_CrO_4(aq)_—indicator and 0.22 M AgNO_3(aq)_—titrator, *n* = 3) and sum of Ca^2+^ and Mg^2+^ cation concentration (EDTA method, Eriochrome Black T—indicator and 0.02 M EDTA—titrator, at pH = 10, *n* = 3) were determined in the overlying water from microcosms *S*, *F*, and *I* within 24 h after the end of the experiment. The samples from microcosms *S*, *F*, and *I* were used to assess the influence of pressure on water and sediment parameters. PCP was quantified in 30 water samples from microcosms *N I* and *N II* after exposure to 6000 hPa and in 28 water samples after exposure to 1000 hPa.

### Organic matter determination

The analysis of the total organic carbon (TOC) concentration in the sediments was performed as described in Lewandowski et al. (2014). Sediment samples were oven dried (105 °C, 4 h) and acidified with 1.0 M HCl. Measurement of TOC was performed (*n* = 4) using a vario TOC cube analyzer (Elementar). The content of the total organic matter was determined by loss on ignition (LOI) method after burning dry sediments (*n* = 3) in muffle furnace at 550 °C for 8 h (Heiri et al. 2001). The precision of TOC analysis amounted to ± 0.03% for 1.61% TOC standard (*n* = 9) and ± 0.05% for 5.39% TOC standard (*n* = 13) and linear regression coefficient of TOC amount against the response of the detector amounted to *r*^2^ = 0.9998.

### Analysis of PCP in water

The samples’ preparation by the solid-phase extraction method and analysis by high-performance liquid chromatography (HPLC) were done in accordance with the methodology validated by Kobusińska et al. (2014) and applied by Maciak et al. (2016). Purified extracts of water samples were injected on the chromatography column Hypersil GOLD thermostated at 25 °C and equipped with a UV-Vis detector. Chromatographic analysis was conducted for 25 min under gradient elution method using acetonitrile and Milli-Q water as the mobile phase.

### Validation and quality parameters of PCP analysis

The recovery of PCP was assured based on external standard method and ranged from 90 to 113%. The precision expressed as the relative standard deviation (RSD) of the mean PCP concentration ranged from 1.8 to 4.7% and was set as 5%. Limit of quantification (LOQ) amounted to 15 ng g^−1^. The linearity of the calibration curve, based on 8 points ranged from 6.0 to 500.0 ng cm^−3^, with correlation coefficient of 0.999. The concentration of PCP in all control samples after 8 h of the experiment decreased by a mean of 8%.

### Statistical analysis

The statistical analysis was carried out using non-parametric ANOVA Kruskal-Wallis test using add-in program Real Statistics (Zaiontz 2017) for the Excel 2010 software (Microsoft). The significance level was set as *p* < 0.05. The null hypothesis states that all result (values of PCP concentration and all physicochemical parameters) of all experiments for all samples will be statistically equal to each other.

## Results

### Parameters of the harbor sediments and the overlying water

There was a strong decrease in the content of organic matter (Table 4) after exposure of bottom sediments to 6000 hPa. Comparing the total organic carbon (TOC) concentration after the experiments at 6000 and 1000 hPa, the highest relative difference was found between *I* sorbents (50%), followed by *S* (21%) and *F* (15%) (Table 4). The TOC concentration in *I*, *S*, and *F* sorbents held at 6000 hPa for 24 h decreased by 85, 32, and 27%, respectively (Tables 3 and 4). In the case of ignited sediments held for 24 h at atmospheric pressure, the mean difference was 71% compared with the initial value. For sorbents *S* and *F*, the TOC concentration decreased by 17 and 18% respectively at 1000 hPa (Tables 3 and 4). The loss on ignition (LOI) method gave similar results. The LOI value of *I*, *S*, and *F* sorbents was lower after the hyperbaric exposure (Table 4).

**Table 4:** Physicochemical parameters of the harbor sediments at different pressure conditions

Parameter	Sorbent	8 h		24 h	
		Pressure [hPa]		Pressure [hPa]	
		1000	6000	1000	6000
TOC [%] ± SD	*Sandy* (*S*)	0.89 ± 0.09	0.76 ± 0.07	0.95 ± 0.05	0.75 ± 0.06
	*Fine* (*F*)	1.54 ± 0.09	1.43 ± 0.18	1.63 ± 0.08	1.38 ± 0.07
	*Ignited* (*I*)	0.12 ± 0.01	0.06 ± 0.01	0.13 ± 0.01	0.07 ± 0.01
LOI [%] ± SD	*Sandy* (*S*)	2.80 ± 0.15	2.40 ± 0.27	3.0 ± 0.13	2.40 ± 0.05
	*Fine* (*F*)	4.90 ± 0.63	4.10 ± 0.39	4.90 ± 0.40	3.50 ± 0.24
	*Ignited* (*I*)	0.60 ± 0.03	0.30 ± 0.07	0.60 ± 0.05	0.30 ± 0.09
LOI/TOC	*Sandy* (*S*)	3.10	3.20	3.20	3.20
	*Fine* (*F*)	3.20	2.90	3.00	2.50
	*Ignited* (*I*)	5.0	5.0	4.60	4.30
Conductivity [mS cm^−1^] ± SD	*Sandy* (*S*)	2.35 ± 0.08	1.41 ± 0.12	2.05 ± 0.09	0.60 ± 0.07
	*Fine* (*F*)	2.25 ± 0.07	2.18 ± 0.02	2.41 ± 0.08	0.74 ± 0.05
	*Ignited* (*I*)	2.72 ± 0.15	1.91 ± 0.09	2.42 ± 0.12	0.90 ± 0.10
pH ± SD	*Sandy* (*S*)	7.0 ± 0.01	7.03 ± 0.01	7.22 ± 0.03	7.22 ± 0.02
	*Fine* (*F*)	7.40 ± 0.04	7.41 ± 0.03	7.53 ± 0.06	7.42 ± 0.04
	*Ignited* (*I*)	7.04 ± 0.04	7.10 ± 0.06	7.13 ± 0.05	7.12 ± 0.06

*TOC* total organic carbon, *LOI* loss on ignition, *SD* standard deviation

The conductivity of sorbents held at atmospheric pressure did not differ among the sorbent groups and did not change after 8 h (number = 18, *p* value = 0.10) (Table 4). This parameter decreased by about 50% after 24 h of the experiment at 6000 hPa compared to results after 8-h exposure (Table 4).

The concentration of Cl^−^, Ca^2+^, and Mg^2+^ ions increased and pH decreased in the seawater of every microcosm at 1000 and 6000 hPa compared with the control samples. The highest concentration of Cl^−^ ion was determined in the water from microcosm containing *F* sorbent and the lowest from microcosm with *I* sorbent. The 24-h exposure of microcosms to 6000 hPa resulted in lower chloride ion concentration in seawater compared to the results after 8 and 24 h at 1000 hPa. The highest increase of Ca^2+^ and Mg^2+^ concentration was noted in the overlying water from *I* microcosm (Table 5). The sum of these ions in the waters from microcosms exposed to the increased pressure was lower than in the waters held at 1000 hPa. The results of conductivity measurement confirmed the results of ion concentration analysis. The pH of water was slightly alkaline during experiment (Table 5).

**Table 5:** Physicochemical parameters of the overlying water from *S*, *F*, and *I* microcosms at different pressure conditions

Parameter	Overlying water from microcosm	8 h		24 h	
		Pressure [hPa]		Pressure [hPa]	
		1000	6000	1000	6000
Cl^−^ [mg dm^−3^] ± SD	*Sandy* (*S*)	3824 ± 46	NDA	3850 ± 113	3680 ± 71
	*Fine* (*F*)	4171 ± 95	NDA	4198 ± 60	4025 ± 42
	*Ignited* (*I*)	3759 ± 92	NDA	3741 ± 99	3560 ± 78
Ca^2+^, Mg^2+^ [mg dm^−3^] ± SD	*Sandy* (*S*)	686 ± 12	NDA	660 ± 15	567 ± 15
	*Fine *(*F*)	662 ± 11	NDA	650 ± 11	604 ± 10
	*Ignited* (*I*)	792 ± 13	NDA	765 ± 16	689 ± 16
Conductivity [mS cm^−1^] ± SD	*Sandy *(*S*)	13.39 ± 0.06	NDA	13.83 ± 0.42	13.62 ± 0.23
	*Fine *(*F*)	14.12 ± 0.38	NDA	14.68 ± 0.43	13.86 ± 0.36
	*Ignited* (*I*)	13.18 ± 0.16	NDA	13.75 ± 0.24	12.84 ± 0.20
pH ± SD	*Sandy* (*S*)	7.42 ± 0.02	NDA	7.73 ± 0.04	7.53 ± 0.04
	*Fine* (*F*)	7.60 ± 0.02	NDA	7.54 ± 0.09	7.25 ± 0.03
	*Ignited* (*I*)	7.70 ± 0.02	NDA	7.76 ± 0.02	7.61 ± 0.05

*SD* standard deviation, *NDA* no data available

### Parameters of *N I* and *N II* sorbents

There was no observable influence of the pressure increased to 6000 hPa on the initial content of organic matter in *N I* and N II^*^ sorbents (Tables 1 and 6 and ^*^information in the text).

**Table 6:** Physicochemical parameters of the Gulf of Gdańsk sediments at different pressure conditions

Parameter	Sorbent	8 h	
		Pressure [hPa]	
		1000	6000
LOI [%] ± SD	*Natural I* (*N I*)	0.60 ± 0.06	0.60 ± 0.06
	*Natural II* (*N II*)	3.00 ± 0.14	2.90 ± 0.29
Conductivity [mS cm^−1^] ± SD	*Natural I* (*N I*)	0.33 ± 0.02	0.37 ± 0.02
	*Natural II* (*N II*)	0.35 ± 0.01	0.44 ± 0.03

*LOI* loss on ignition, *SD* standard deviation

The conductivity of sediments from the Gulf of Gdańsk held at 1000 hPa decreased when compared to the initial value (Tables 1 and 6). After the hyperbaric exposure, value of this parameter for *N II* sorbent was higher than that for *N II* sorbent held at atmospheric pressure (Tables 1 and 6).

### Concentration of PCP in *N I* and *N II* microcosms

**Fig. 3:**
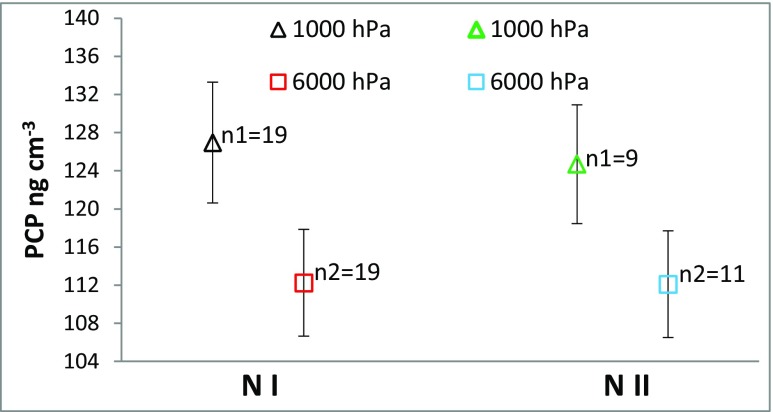
The concentration of PCP in the overlying water after the 8-h experiment at 1000 and 6000 hPa. *N I* Natural I, *N II* Natural II, *n1* number of the results for experiment at 1000 hPa, *n2* number of the results for the experiment at 6000 hPa. Error bars denote ± 5% RSD

The sorption of PCP occurred at pH ≈ 8 (Table 7). On the basis of all results (*n* = 58), it was found that after 8-h exposure of *N I* (*n* = 19) and *N II* (*n* = 11) microcosms to 6000 hPa, the concentration of PCP in the overlying water was statistically significantly lower (*p* value = 0.001) when compared to *N I* (*n* = 19) and *N II* (*n* = 9) microcosms held at 1000 hPa (Fig. 3). Average difference between concentration of PCP in every microcosm held at higher pressure and every microcosm held at atmospheric pressure was 8%.

**Table 7:** Physicochemical parameters of the overlying water from *N I* and *N II* microcosms at different pressure conditions

Parameter	Overlying water from microcosm	8 h	
		Pressure [hPa]	
		1000	6000
Conductivity [mS cm^−1^] ± SD	*Natural I* (*N I*)	11.35 ± 0.15	11.39 ± 0.06
	*Natural II* (*N II*)	11.54 ± 0.13	11.70 ± 0.12
pH ± SD	*Natural I* (*N I*)	7.90 ± 0.01	7.90 ± 0.01
	*Natural II* (*N II*)	7.90 ± 0.01	7.90 ± 0.01

*SD* standard deviation

## Discussion

### Influence of the increased pressure on the sediment parameters

The sorption/desorption processes of a given substance depend on the characteristics of mineral structure, the type of organic matter, and the characteristic of water reservoir (LeBoeuf and Weber 1997; Chorover and Brusseau 2008; Niemirycz and Jankowska 2011). The changes of the physicochemical parameters of the sediments after exposure to the increased pressure probably also depended on these factors (Tables 4 and 5).

The initial LOI/TOC ratio was 2.10, 2.80, and 3.10 for *I*, *S*, and *F* sorbents, respectively (Table 3). Usually, the LOI to TOC conversion factor is in the range of 1.70–2.0 (Veres 2002; Rallakis et al. 2013). It is interesting to note that for *F* sediments after an 8-h hyperbaric exposure, this factor reached 2.90 and dropped significantly to 2.50 after 24 h (Table 4). The cause of this could be a slower process of stabilization (within 24 h) due to the *F* sediments containing an increased amount of the fine fraction (*Φ* < 0.063 mm) from the Port of Gdynia. Taking into account all the parameters of the bottom sediments, the highest difference between 8- and 24-h experiments was noted for *F* sorbent after exposure to the increased pressure.

The possible cause of the decrease of LOI and TOC values of *S*, *F*, and *I* sorbents after the first part of experiment, carried out at atmospheric pressure, could be the method of sediment preparation by initial air drying and rewetting. During such a procedure, the hydrogen bonds in the organic matter are broken. That leads to the destabilization of some structures of the organic matter and to the stronger dissolution of matter contained in the sediment (Birch 1959; Raveh and Avnimeleh 1978). The storage time (3 years) could also exert its effect on the harbor sediments. After the experiment with sorbents prepared using the sediments from the Gulf of Gdańsk, organic matter content was not affected even by the hyperbaric exposure (Table 6).

Beside the type of bottom sediments, the depth and environment from which sediments were sampled also seem to be important. The material for *S*, *F*, and *I* sorbents was silty sediments, originating from a depth of 10 m in the Port of Gdynia, where hydrostatic pressure is five times lower than that during experiment. Moreover, sediments in this disturbed benthic environment are subjected to regular intensive mixing by ships. Probably, this is why organic matter decreased even after atmospheric exposure. The material for *N I* and *N II* sorbents was sandy sediments, collected from less polluted environment of the Gulf of Gdańsk. The sampling depth was 40 m where pressure was 5000 hPa, close to that in the experiment, resulting in stable organic matter under applied conditions.

The increase of overall pressure could lead to increase in the solubility of minerals, allowing the water to reach the saturation point faster, which leads to the process of crystallization (Stoch 1974). Probably, this explains why conductivity of the harbor sediments was lower after experiment in the hyperbaric chamber (Table 4).

The hyperbaric exposure of the harbor sediments could be referred to the routine dredging works carried out in ports to deepen the bottom channels. According to the guidelines (HELCOM Guidelines 2007), the dredged uncontaminated material of appropriate diameter can be disposed in a selected area in the sea (dumping site), often at a different depth to the depth at which dredging works took place. In the case of the Port of Gdynia, where samples of sediments where collected, dumping site is located at a depth of 20–50 m (Michałek et al. 2014), where the total pressure reaches 3000 to 6000 hPa. Considering this, it is possible that observed changes of *S*, *F*, and *I* sorbent parameters are occurring in the natural environment. The results of this study can be taken into consideration by other researchers examining the areas of the dumping sites.

### Influence of the increased pressure on the water parameters

The phenomenon of lowering Cl^−^ ion concentration due to the influence of pressure force was observed in the first works describing extraction of the interstitial waters from sediments (Shishkina 1972; Lindström and Bloomquist 1980). The method involved using pressure 100 times greater than in the present work. Other researchers (Parashiva-Murthy and Perrel 1972), which applied pressure (6895 hPa) close to the one which was simulated inside the hyperbaric chamber, observed lowering of Ca^2+^ ions and increase of Mg^2+^ ions after squeezing the pore waters. Ions of Mg^2+^, K^+^, and also Fe^2+^ are the most mobile (Stoch 1974). Radke et al. (2012) revealed that the exposure of bottom sediments from the Port of Gdynia to the temperatures between 400 and 600 °C resulted in decarbonization of very fine-grained matter with magnesium carbonate. This can be the reason why the highest concentration of Ca^2+^ and Mg^2+^ was found in the water overlying the ignited harbor sediments. The dissolving of the organic matter and the hydronium ion transfer from minerals surface (Stoch 1974) could have an impact of lowering the water pH (Table 5).

The changes of the physicochemical parameters of the sediments and the water, which affect sorption, should not take place during such an experiment. This indicates that the initial hyperbaric exposure (before experiment) of microcosms could be necessary.

### Influence of hydrostatic pressure on the sorption process

Hydrostatic pressure is presented in the scientific literature as one of the parameters which may, among others, influence the sorption/desorption processes (Grossl et al. 1997; Bigus et al. 2014) or increase/decrease growth of bacteria population (Somero 1992; Schedler et al. 2014; Scoma et al. 2016). However, there are not many laboratory studies examining the sorption of persistent organic pollutants (POPs) at a pressure higher than atmospheric. The pressure at bottom of the sea, in conditions of limited depth, may contain, in addition to the hydrostatic component, also hydrodynamic component of an oscillatory nature due to the presence of wind waves (Cieślikiewicz and Badur 2013). We estimate, however, that at a depth of 50 m, even in conditions of high winds, the hydrodynamic part of pressure varies within ± 2% of atmospheric pressure, i.e., ± 20 hPa. With the assumed pressure of 6000 hPa, this is a negligible force that we ignore in this work.

The pH is an important factor in the sorption and dissolution processes of PCP. The pH ≈ 8 of the overlying water means that PCP existed in an anionic form, which results in reduced hydrophobicity and limited sorption/desorption efficiency in humus or sediments (DiVincenzo and Sparks 2001; Site 2001). In laboratory studies concerning partitioning of PCP between solid and liquid phases, an 8-h period was sufficient to reach an equilibrium concentration (Site 2001; Chen et al. 2004; Subramanian et al. 2010; Liping et al. 2015). The influence of simulated hydrostatic pressure on the physicochemical parameters of *N I* and *N II* sorbents and its overlying water was minor or not significant (Tables 6 and 7) compared to *S*, *F*, and *I* sorbents (Tables 4 and 5). Based on the available data, it was assumed that the sorption process of PCP was occurring in stable conditions. The PCP concentrations in the overlying water of the microcosms containing *N I* and *N II* sorbents exposed in the experiment to the same pressure were compared (Fig. 3). The amount of fine fraction (1 or 25%) and organic matter (0.60 and 2.95% after the experiment) did not significantly affected the sorption process in the present study in accordance with findings of other papers (DiVincenzo and Sparks 2001; D’Angelo and Reddy 2003). Considering the specific nature of the harbor bottom sediments, we can determine that the hydrostatic pressure at 50 m appears to be inconsequential in the sorption process of PCP (Fig. 3).

According to the mass balance concept, the amount of substance that has entered the reservoir is equal to the amount that has left the reservoir and been accumulated in it. If the sorption process in bottom sediments is affected by hydrostatic pressure, higher than that in the experiment, it must also change with depth. In situation where influence of water and sediment parameters (Bigus et al. 2014) is negligible, for some of the substances, it may be easier to leave the reservoir or stay longer in it at different depths.

## Conclusions

Hydrostatic pressure is an important, but scarcely studied parameter in the persistent organic pollutant sorption and desorption processes. The conducted study revealed complexity of all reactions occurring in the microcosms during the experiments. The pressure of 6000 hPa, corresponding to the depth of 50 m, had minor influence on the sorption of pentachlorophenol in the marine bottom sediments. No increase in PCP sorption in marine sediments is disadvantageous, since PCP and related compounds are then dissociated and/or suspended in the water column. Thus, they remain bioavailable for longer and can be absorbed by organisms at different trophic chain levels resulting in bioaccumulation and biomagnification. It is a subject of particular concern as the studied coastal waters represent a frequently visited recreation, seaside area.

The simulated pressure of 6000 hPa is relatively low in relation to the possible hydrostatic pressure that can be noted on the bottom of the deeper seas and oceans. Thus, the results presented in this study can provide only some new insight into the importance of hydrostatic pressure in the sorption/desorption processes, which occur on the seabed of the ocean systems.
